# Provider perceptions of severe pediatric traumatic brain injury care priorities across hospitals in South America before and during the COVID-19 pandemic

**DOI:** 10.1371/journal.pone.0275255

**Published:** 2022-09-29

**Authors:** Shyam J. Deshpande, Julia Velonjara, Silvia Lujan, Gustavo Petroni, Jin Wang, Kushang V. Patel, Linda Ng Boyle, Michael J. Bell, Monica S. Vavilala, for the PEGASUS Argentina Study Group

**Affiliations:** 1 Department of Anesthesiology & Pain Medicine, University of Washington, Seattle, WA, United States of America; 2 Department of Pediatrics, University of Washington, Seattle, WA, United States of America; 3 Harborview Injury Prevention & Research Center, Seattle, WA, United States of America; 4 Centro de Informática e Investigación Clínica, Rosario, Argentina; 5 Department of Industrial and System Engineering, University of Washington, Seattle, WA, United States of America; 6 Children’s National Hospital Critical Care Medicine, Washington, DC, United States of America; CHU Nantes: Centre Hospitalier Universitaire de Nantes, FRANCE

## Abstract

**Background:**

To understand provider perceptions of the COVID-19 pandemic on priorities of severe pediatric traumatic brain injury (TBI) care across hospitals in South America.

**Methods:**

Site principal investigators (PIs) from 17 hospitals in South America enrolled in the PEGASUS-Argentina randomized controlled trial completed questionnaires regarding order of tasks performed in the care of a typical pediatric patient with severe TBI before (2019) and during (2021) the COVID-19 pandemic. Acute care processes were examined by quintiles to identify early, mid, and late actions and were categorized and compared. Associations of hospital volume and subspecialty resource availability with prioritization of key process actions were examined.

**Finding:**

Site PIs from 15 and 16 hospitals completed the surveys in 2019 and 2021, respectively, including 14 who completed both. Action category order was stable between 2019 and 2021 and were ranked in priorities as: initial encounter, primary survey, interventions and invasive monitors, diagnostics, medications, staff communication, then disposition (in 2019) or nutrition (in 2021). There was variation in specific action order between hospitals at both timepoints, with only a few initial encounter and disposition actions limited to a single quintile. There was no reported association between hospital volume or subspecialty resource availability with prioritization of key process actions.

**Interpretation:**

Despite novel healthcare challenges presented by the COVID-19 pandemic, providers in South America perceived maintaining standard severe pediatric TBI care consistent with BTF guidelines. There was large variability in specific action order between individual hospitals reported.

## Introduction

Traumatic brain injury (TBI) is a common cause of pediatric morbidity and mortality worldwide, affecting more than three million children annually [1]. The most common causes of pediatric TBI globally are falls and motor vehicle crashes, resulting in a bimodal age distribution, with highest rates between 0–3 and 15–18 years of age. Globally pediatric TBI disproportionately affects males, children with low socioeconomic status [1], and in the US children with preexisting comorbidities [2]. There is a paucity of TBI epidemiologic data in Latin America [3], but previous studies have shown that TBI disproportionately affects people of mixed and indigenous race compared to white counterparts in Latin America [4]. While a majority of pediatric TBI cases are mild and managed nonoperatively, severe pediatric TBI (Glasgow Coma Scale (GCS) ≤ 8) accounts for 3–7% of cases globally [1] and carries a 14–21% mortality rate [5]. Pediatric TBI results in deficits with complex pathophysiological processes, including long-term cognitive[6], health, and social deficits [7]. And, these injuries are responsible for significant resource utilization, costing more than $1 billion annually [8].

The Brain Trauma Foundation (BTF) has published serial guidelines on the acute care management of infants and children with severe TBI [9, 10]. The 2019 guidelines focus on three primary therapeutic targets, including prevention and management of intracranial hypertension, optimization of cerebral perfusion pressure, and optimization of partial pressure of brain tissue oxygen, as well as treatment of acute cerebral herniation, and the utility of decompressive craniectomy, barbiturate infusion, hypothermia, hyperventilation, and hyperosmolar therapies. Although most studies included in the guidelines originated from the U.S., this compendium remains the most authoritative guide for the acute care of children with severe TBI [9].

The Pediatric Guideline Adherence and Outcomes (PEGASUS) program is an implementation science-based program for children with severe TBI, which has been pilot tested in the U.S [11, 12]. Implementation of the PEGASUS program not only resulted in increased adherence to key performance indicators (KPI) but also demonstrated significant improvements in survival and favorable discharge disposition [12]. Furthermore, implementation of the PEGASUS program did not result in increased hospital costs, making it a cost-effective program [13]. The National Institutes of Health PEGASUS-Argentina randomized controlled trial (RCT) was funded in 2019 to test PEGASUS program effectiveness in improving pediatric TBI guideline adherence in South America [14]. Importantly, there is a paucity of evidence surrounding pediatric TBI care from South America used in clinical guidelines; an overwhelming majority of the studies used in the BTF evidentiary tables are from North America and Europe [9], with only three studies originating from South America [15–17].

In early 2020, the SARS-CoV-2 virus was first confirmed in Latin America, and by the Spring, Latin America had become an epicenter of the disease [18]. Hospitals participating in the PEGASUS-Argentina RCT in Argentina, Paraguay, and Chile reported a steep rise in COVID-19 cases and deaths between April and June 2021 [19, 20]. Already strained, health care systems in South America faced new pediatric care threats to capacity and infrastructure [21], economic impact on child health [22], barriers to routine childhood care [23], severe manifestations of pediatric COVID-19 [24], and likely caregiver loss [25]. Furthermore, preventative lockdown policies negatively impacted pediatric health and contributed to economic strain [26]. The effect of the COVID-19 pandemic on pediatric TBI care has not been described but is important to understand. The specific aims of this study were to: 1) Compare processes of care for children with severe TBI before and during the COVID-19 pandemic, 2) Assess variability in process order between hospitals before and during the COVID-19 pandemic, and 3) Determine hospital characteristics associated with prioritization of key BTF-guideline-driven processes.

## Methods

The PEGASUS-Argentina study is an implementation science RCT to enhance compliance with published TBI guidelines. This study is a prospective, longitudinal, descriptive examination of the PEGASUS-Argentina RCT to determine the reported processes of severe pediatric TBI care in participating hospitals at two time points, in 2019 and 2021, before and during the COVID-19 pandemic.

We also initially planned to have on-site research assistants collect prospective, time-stamped, patient level data on type and timing of processes of care children with severe TBI at each hospital; yet, non-essential personnel (research assistants) were restricted from clinical settings after the onset of the COVID-19 pandemic. Therefore, we had to adapt data collection methodology. Site principal investigators (PI) for each of the 17 hospitals completed questionnaires regarding the order of tasks performed at their institution in the care of a typical patient presenting to their pediatric intensive care unit (PICU) with severe TBI, using the following case description:

“Consider a severe (GCS 3–8) TBI pediatric patient arriving in your ICU. Keep in mind the most common patient characteristics that you see in your ICU. Consider each action and prioritize when an action may be performed during care… Select ‘not applicable’ for actions that are not available at your site or would not be performed in the case of your typical severe TBI pediatric patient.”

Surveys at both timepoints were conducted in Spanish and translated to English. Based on local preference, the first questionnaire in August 2019 elicited in-person hand-completed responses, allowing respondents to rank up to 50 predefined actions, omit actions not included in their typical process flow, rank events as “ties” if deemed that they occurred concurrently, and write in additional steps if needed. The second questionnaire sent May-June 2021 was completed via an online questionnaire due to travel restrictions during the COVID-19 pandemic (SurveyMonkey, Momentive Inc., San Mateo, California, USA) and did not allow actions to be ranked as ties.

The 2019 survey was conducted immediately prior to institutions collecting PEGASUS-Argentina baseline data. From September 2019-September 2020, all participating sites collected prospective patient data without any trial interventions. By the time the 2021 survey was conducted, the sites within the intervention arm of the PEGASUS-Argentina RCT (8 of 17) had received approximately 6 months of intervention implementation. One site included in 2019 had no patients eligible for the main RCT during the baseline period, so was not included in subsequent study stages. Interventions included access to PEGAUS checklist goals for the care, interdisciplinary TBI education, site PI logistical support, and standardized case reviews to identify barriers to guideline adherence.

### Categorizing acute TBI care

Reported acute processes of TBI care were ranked sequentially to account for skipped steps on the written survey. Processes were examined by quintiles to account for differences in denominator (as the number of actions for each hospital varied) in order to identify early, mid, and late actions. Actions were also categorized into eight groups to compare overall flow of TBI care: initial encounter, primary survey, interventions and invasive monitors, diagnostics, medications, staff communication, disposition (i.e. transfer or discharge), and nutrition. Categories were predefined during survey development.

### Statistical analysis

Hospital characteristics are described as counts and percentages. As previous studies have indicated that higher volume PICUs have better outcomes [27–29], we examined whether hospital volume and subspecialty resource availability were associated with prioritization of key BTF guideline-driven process actions using linear regression. Key process actions examined included actions with BTF guideline Level 2 evidence or greater as well as actions listed within BTF baseline care guidelines. These included “hypertonic saline given,” “intracranial pressure (ICP) monitor placement,” “computed tomography (CT) ordered,” “arterial line placement,” and “sedation.”

Using sequentially ranked survey data grouped into quintiles as described above, we generated heat maps to compare order of actions across and between hospitals, with individual hospitals on the x-axis, actions grouped by category on the y-axis, and box fill grayscale representing quintile. Heat maps were then inspected qualitatively for changes in pattern, and mean percentile of actions within each category were determined to compare overall process of care between survey periods.

Again using sequentially ranked survey data grouped into quintiles, we then generated bubble plots to examine the variability of actions across hospitals, with quintile on the x-axis, actions grouped on the y-axis, and bubble size representing the number of hospitals ranking an action at each quintile. Bubble plots were inspected qualitatively to determine whether an action was isolated to a single quintile (i.e. there was concordance between hospitals when an action occurred) or spread across quintiles (i.e. an action occurred early in some hospitals’ process of care but late in others’). Data were processed using R Version 3.6.1 [30] and visualized using *ggplot2* package [31].

This study was approved by the institutional review boards at each of the PEGASUS-Argentina study hospitals and by the institutional review board of the University of Washington.

## Results

Characteristics of the 17 participating hospitals are given in Table 1; the majority are provincial hospitals located in Argentina. Hospitals serve a median population of approximately 660,000 individuals, have 14 PICU beds, and see 15 cases of severe pediatric TBI annually. Most hospitals have public and private emergency services available, and patients typically arrive by ambulance. Approximately 2/3 of hospitals have a neurosurgeon on-call and 1/3 have a neurosurgeon present on-site 24 hours per day. All hospitals have capacity for CT imaging 24 hours per day and ICP monitoring, but only 1/3 have magnetic resonance imaging (MRI) capacity.

**Table 1:** Hospital characteristics for the 17 participating hospitals in South America.

Characteristic	Level	Overall
		n = 17
**Hospital characteristic**		
Country	Argentina	15 (88.2)
	Chile	1 (5.9)
	Paraguay	1 (5.9)
Hospital dependency	Provincial	12 (70.6)
	Provincial + National	2 (11.8)
	Municipal	2 (11.8)
	Private	1 (5.9)
**Hospital impact**		
Population covered		660,000 (110,000)
Total beds		165 (121)
Pediatric intensive care unit beds		14 (4)
Severe pediatric traumatic brain injury cases		15 (11)
**Emergency medical services available to hospital**		
Private + Public		11 (64.7)
Public		5 (29.4)
Private		1 (5.9)
**Patient arrival method to hospital (n = 17)**		
Ambulance	0–25%	1 (5.9)
	26–50%	4 (23.5)
	51–75%	1 (5.9)
	76%-100%	10 (58.8)
	Unknown	1 (5.9)
Car	0–25%	9 (52.9)
	26–50%	4 (23.5)
	51–75%	0
	76%-100%	0
	Unknown	4 (23.5)
Other	0–25%	14 (82.4)
	26–50%	0
	51–75%	0
	76%-100%	1 (5.9)
	Unknown	2 (11.8)
**Hospital resources available**		
Neurosurgery	Yes–a neurosurgeon is on call	11 (64.7)
	Yes–a neurosurgeon is present on the site 24 hours	6 (35.3)
CT	Yes–available 24 hours per day	17 (100.0)
MRI	Referral to other centers for MRI	8 (47.1)
	Yes–available 24 hours per day	7 (41.2)
	No	2 (11.8)
ICP monitoring	Yes, Fiber Optic	16 (94.1)
	Yes, Electronic Sensor	1 (5.9)

Number of severe pediatric traumatic brain injury cases from 1^st^ year of study period reported. CT = computed tomography. MRI = magnetic resonance imaging. ICP = intracranial pressure. Continuous variables = median (IQR). Categorical variables = number (%).

**Fig 1:**
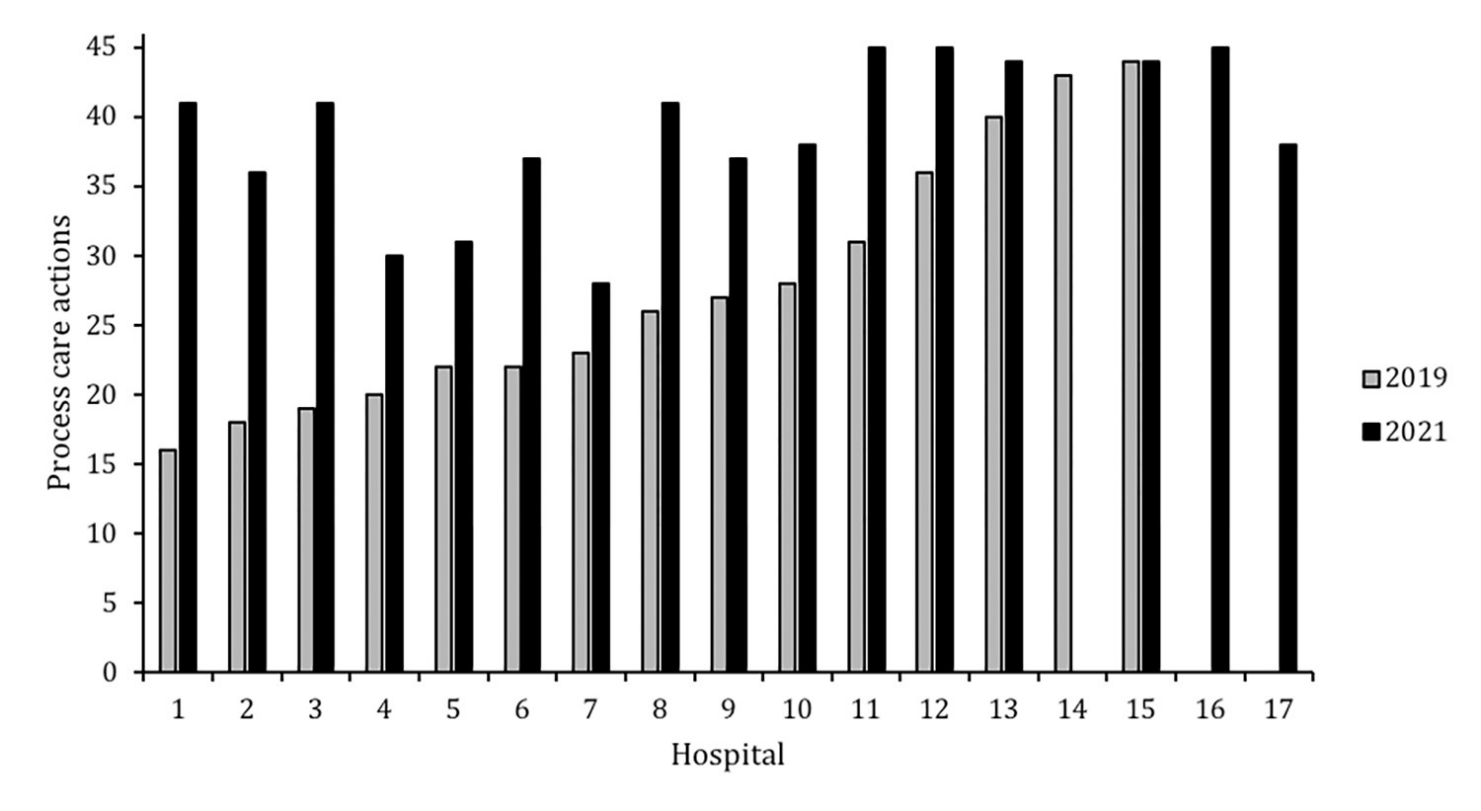
Variation in number of severe pediatric traumatic brain injury process care actions across 17 South American hospitals at two timepoints: 2019 and 2021.

Site PIs from 15 and 16 hospitals completed the survey in 2019 and 2021, respectively, including 14 who completed both (Fig 1). Forty-six predefined actions were included in the final analysis (four excluded due to redundancy). Site PIs ranked an average of 28 actions (range 16–44) in their hospital’s process in 2019 and 39 (range 28–45) in 2021. Of the 14 site PIs who participated in both surveys, 13 (92.9%) reported an increase in number of process actions, 1 (7.1%) reported the same, and zero reported a decrease.

**Fig 2:**
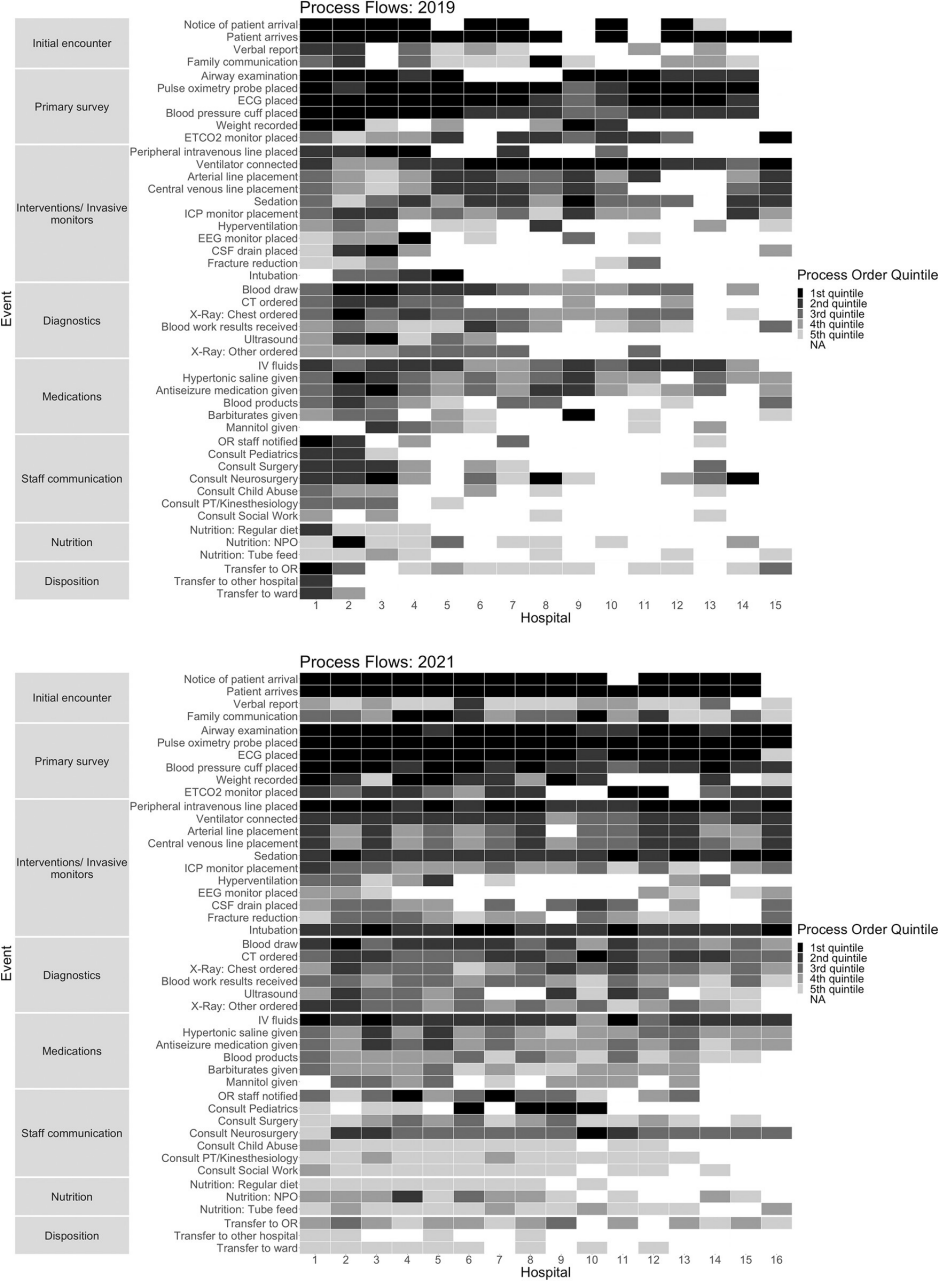
Heat maps of hospital process order in severe pediatric traumatic brain injury care in 2019 (a) and 2021 (b). Hospitals on x-axis. Events grouped by category on y-axis. Raw survey data for each hospital ranked in sequential order to account for survey responses that skipped steps. Ties reported as the same rank with subsequent rank numbers accounting for the number of ties. Rank order batched into quintile order for each hospital. Box grayscale = order quintile of each event for a hospital. Empty boxes = events not included in a hospital’s survey response. ECG = electrocardiogram. ETCO2 = end tidal carbon dioxide. ICP = intracranial pressure. EEG = electroencephalogram. CSF = cerebrospinal fluid. CT = computed tomography. IV = intravenous. OR = operating room. PT = physical therapy. NPO = nil per os.

Process order for 2019 and 2021 is shown in Fig 2A and 2B, respectively. Reported action categories order was stable between 2019 and 2021, prioritized with average action order percentiles as: initial encounter (12^th^ & 14^th^), primary survey (20^th^ & 17^th^), interventions and invasive monitors (48^th^ & 40^th^), diagnostics (55^th^ & 50^th^), medications (58^th^ and 67^th^), staff communication (60^th^ & 83^rd^), then disposition (81^st^ & 93^rd^) or nutrition (93^rd^ & 84^th^).

**Fig 3:**
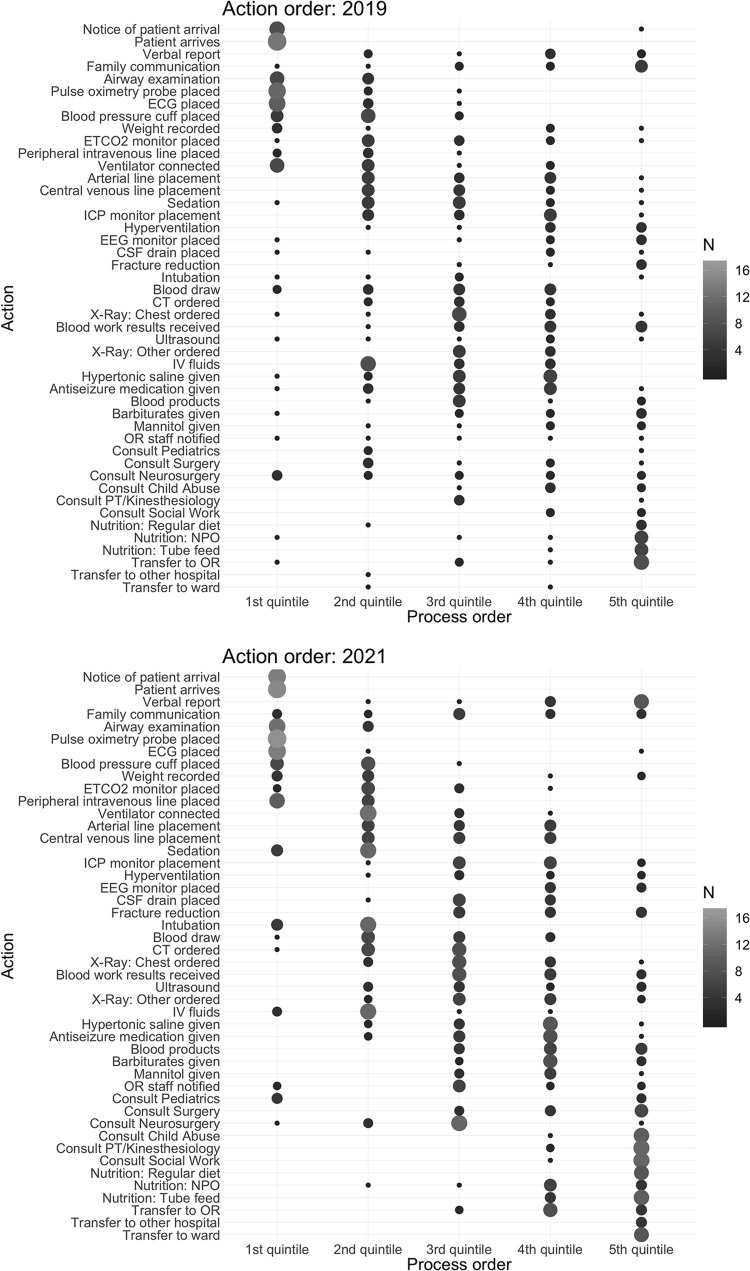
Variability of specific action order in severe pediatric traumatic brain injury care across South American hospitals in 2019 (a) and 2021 (b). X-axis represents earliest to latest actions reported. Larger bubble size and lighter grayscale represent greater occurrence of hospitals ranking an action in a specific quintile. ECG = electrocardiogram. ETCO2 = end tidal carbon dioxide. ICP = intracranial pressure. EEG = electroencephalogram. CSF = cerebrospinal fluid. CT = computed tomography. IV = intravenous. OR = operating room. PT = physical therapy. NPO = nil per os.

There was large variability in specific action order between hospitals; only a few specific initial encounter and disposition actions were limited to a single quintile (Fig 3A and 3B). Hospitals generally agreed on actions that occurred at the very beginning (arrival, basic vital signs) or very end (patient disposition) of their processes; actions outside of these extremes generally spanned multiple quintiles. In both survey years, “patient arrives” was isolated to the first quintile of process order, whereas “family communication” spanned all five quintiles of process order. When examining variability of key process actions, there was no association between hospital volume (number of PICU beds, number of severe pediatric TBI cases) or subspecialty resource availability (presence of on-call versus 24 h in-house neurosurgery) with prioritization of “hypertonic saline given,” “ICP monitor placement,” “CT ordered,” “arterial line placement,” or “sedation” (S1 Table).

## Discussion

The main purpose of this study was to understand the provider perception of the COVID-19 pandemic on pediatric TBI care. Our data show that reported priorities of severe pediatric TBI care in South America remained stable from 2019 pre-COVID-19 pandemic to 2021 during the COVID-19 pandemic. At both timepoints, hospitals prioritized the primary trauma survey, airway management, placement of necessary lines, and diagnostic studies, followed by medication administration, consultation of specialty services, and non-OR disposition. Our findings indicate that providers reported maintaining most of the high priorities in severe pediatric TBI care despite COVID-19 pandemic related healthcare challenges.

The COVID-19 pandemic has strained trauma care globally, limiting hospital beds and hospital staff availability, complicating airway management procedures, and drastically reducing blood product supplies [32]. The COVID-19 pandemic especially stressed the pre-hospital medical system, necessitating stringent patient severity triage, restricting availability of hospitals to receive patients [25], and creating longer response times [33]. It is possible that hospitals in this study were able to maintain priorities of care because these patients were the most critically injured/ill, there were a small number of severe pediatric TBI admissions which had a relatively minor impact on hospital resources, because the COVID-19 pandemic may have strained resources for respiratory illness rather than TBI, or through deliberate institutional systems for maintaining trauma care during the COVID-19 pandemic [34]. Our work adds to the understanding of how clinicians perceived severe pediatric TBI care in South America during the COVID-19 pandemic.

While overall categories of actions were performed in a similar order between institutions, there was large variability in specific action order between individual hospitals. This finding is consistent with prior work [35]. Vavilala and colleagues found that PICU care of children with severe TBI varies significantly, with KPI adherence varying between 55.6 to 83.7% across seven pediatric trauma centers in Argentina. The variation described here may be due to differences in institutional resources, hospital culture, faculty and staff factors, or heterogeneity of patient population. Observing variation in care is also an opportunity for optimization of processes across multiple hospitals. Multiple studies have demonstrated guideline-based internal institutional standardization improves outcomes for children with severe TBI [11, 36, 37]. Pineda and colleagues showed that implementation of a time-sensitive, severity-based pediatric neurocritical care program improved Glasgow Outcome Scale and favorable disposition [36]. O’Lynnger and colleagues similarly showed improvement in hospital disposition and mortality after implementation of a multidisciplinary, stepwise protocol based on BTF guidelines [37]. Standardization of severe pediatric TBI care may improve guideline adherence and outcomes across hospitals currently enrolled in the intervention arm of the PEGASUS-Argentina RCT. For example, reducing variability in the time of invasive vascular or cerebral monitor placement may facilitate more efficient administration of ICP-targeted therapies.

There was no association between hospital volume (number of PICU beds, number of severe pediatric TBI cases) or subspecialty resource availability (presence of on-call versus 24 h on-site neurosurgery) with prioritization of key process actions (“hypertonic saline given,” “ICP monitor placement,” “CT ordered,” “arterial line placement,” and “sedation”). Previous studies generally indicate that higher volume PICUs have better outcomes [27–29], although this is controversial [33, 38]. It is possible that higher volume centers implement other interventions that improve outcomes outside of prioritization of the key process actions measured in this study. However, our finding may be due to limitations of our survey format; in future work it may be possible to detect associations of hospital characteristics with prioritization of key process actions, using patient-level time-stamped continuous data as opposed to ordinal survey responses.

This study has some strengths and limitations. Strengths are study of detailed reports of acute care processes relevant to TBI care and outcomes, as well as the availability of pre-COVID-19 pandemic data. Limitations include self-report and risk of respondent recall; we intended to collect prospective time-stamped, patient-level data, which would provide more accurate evaluation of process flows and insight on timing (including bottlenecks and rate determining steps). However, due to restrictions of non-essential personnel in clinical settings during the COVID-19 pandemic, we were unable to have research assistants conduct in person evaluations using previously developed technology to directly observe clinical care at these time points [39–40]. We also recognize that the experiences of these 17 hospitals (of which a majority are from Argentina) may not represent the experiences of institutions across other South American countries. Additionally, participation in the PEGASUS-Argentina RCT may have externally bolstered hospitals’ maintenance of priorities of care, yet participation does not obviate the additional health care barriers faced during the COVID-19 pandemic. The increase from 2019 to 2021 in number of actions ranked is most likely a limitation of the change in survey format (i.e. in-person hand-completed versus electronic) or due to a survey training effect (i.e. site PIs were able to provide more thorough answers the to the 2021 because they knew what was going to be asked and had time to prepare). In spite of these limitations, our work provides new information on the effect of the COVID-19 pandemic on order and processes of severe pediatric TBI care in South America.

## Conclusions

Despite the COVID-19 pandemic, providers in South America reported maintaining overall priorities of severe pediatric TBI care from 2019 pre-COVID-19 pandemic to 2021 during the COVID-19 pandemic. There was substantial inter-hospital variation in number of process steps and specific action order at both timepoints. Future work should focus on examining patient-level TBI care and outcome data to confirm provider perceptions.

## Supporting information

S1 Table Lack of association between hospital volume and subspecialty resource availability with order percentile of key process actions in 2019 and 2021 during severe pediatric traumatic brain injury (TBI) care. Results reported as linear regression ß coefficient (95% CI). Neurosurgery on-call versus in-person 24 hours per day. sTBI = Cases of severe pediatric TBI per year. PICU = hospital PICU beds. ICP = intracranial pressure, CT = computed tomography.
(DOCX)

S1 Data (XLSX)
