# Supplementary material for: Provider perceptions of severe pediatric traumatic brain injury care priorities across hospitals in South America before and during the COVID-19 pandemic
Source: PLoS One. 2022 Sep 29;17(9):e0275255. doi: 10.1371/journal.pone.0275255 (PMC9522258; doi:10.1371/journal.pone.0275255)
Supplement: S1 Table — Results reported as linear regression ß coefficient (95% CI). Neurosurgery on-call versus in-person 24 hours per day. sTBI = Cases of severe pediatric TBI per year. PICU = hospital PICU beds. ICP = intracranial pressure, CT = computed tomography. (DOCX) [file pone.0275255.s001.docx]

**Supporting information**

**S1 Table.** **Lack of association between hospital volume and subspecialty resource availability with order percentile of key process actions in 2019 and 2021 during severe pediatric traumatic brain injury (TBI) care.**

|  | **Hypertonic saline administration** | | **ICP monitor placed** | | **CT ordered** | | **Arterial line placement** | | **Sedation** | |
| --- | --- | --- | --- | --- | --- | --- | --- | --- | --- | --- |
|  | 2019 | 2021 | 2019 | 2021 | 2019 | 2021 | 2019 | 2021 | 2019 | 2021 |
| **Neurosurgery** | 0.119  (-0.133, 0.370) | -0.077  (-0.285, 0.131) | -0.005  (-0.255, 0.244) | 0.036  (-0.144, 0.215) | 0.058  (-0.243, 0.360) | 0.115  (-0.008, 0.239) | 0.056  (-0.241, 0.352) | -0.005  (-0.177, 0.167) | -0.093  (-0.351, 0.166) | -0.002  (-0.084, 0.080) |
| **Severe TBI** | -0.006  (-0.015, 0.003) | 0.016  (-0.007, 0.038) | -0.004  (-0.016, 0.008) | 0.001  (-0.020, 0.022) | 0.009  (-0.011, 0.029) | -0.004  (-0.020, 0.012) | -0.007  (-0.021, 0.007) | 0.010  (-0.010, 0.028) | -0.006  (-0.017, 0.005) | 0.002  (-0.007, 0.010) |
| **PICU** | -0.005  (-0.034, 0.025) | 0.002  (-0.017, 0.020) | -0.000  (-0.027, 0.026) | 0.004  (-0.012, 0.020) | -0.001  (-0.028, 0.025) | 0.002  (-0.011, 0.014) | 0.014  (-0.016, 0.044) | 0.009  (-0.006, 0.023) | 0.012  (-0.011, 0.035) | 0.001  (-0.006, 0.008) |

Results reported as linear regression ß coefficient (95% CI). Neurosurgery on-call versus in-person 24 hours per day. sTBI = Cases of severe pediatric TBI per year. PICU = hospital PICU beds. ICP = intracranial pressure, CT = computed tomography
